# Emergency surgery for splenic flexure cancer: results of the SFC Study Group database

**DOI:** 10.1186/s13017-021-00365-0

**Published:** 2021-04-29

**Authors:** Nicola de’Angelis, Eloy Espin, Frederic Ris, Filippo Landi, Bertrand Le Roy, Federico Coccolini, Valerio Celentano, Angela Gurrado, Denis Pezet, Giorgio Bianchi, Riccardo Memeo, Giulio C. Vitali, Alejandro Solis, Christine Denet, Salomone Di Saverio, Gian Luigi de’Angelis, Miquel Kraft, Paula Gonzálvez-Guardiola, Aine Stakelum, Fausto Catena, David Fuks, Des C. Winter, Mario Testini, Aleix Martínez-Pérez, on behalf of the SFC Study Group

**Affiliations:** 1 Unit of Minimally Invasive and Robotic Digestive Surgery, General Regional Hospital F. Miulli, Acquaviva delle Fonti, Bari, Italy; 2 University of Paris Est, UPEC, Créteil, France; 3 Unit of Colorectal Surgery, Department of General and Digestive Surgery, University Hospital Vall d’Hebron-Universitat Autonoma de Barcelona, Barcelona, Spain; 4 Service of Abdominal Surgery, Geneva University Hospitals and Medical School, Geneva, Switzerland; 5 Department of General Surgery, Viladecans Hospital, Barcelona, Spain; 6 Department of Digestive and Hepato-biliary Surgery, Hospital Estaing, CHU Clermont-Ferrand, Clermont-Ferrand, France; 7 General, Emergency and Trauma Surgery Department, Bufalini Hospital, Cesena, Italy; 8 Minimally Invasive Colorectal Unit, Portsmouth Hospitals NHS Trust, Portsmouth, UK; 9 University of Portsmouth, Portsmouth, UK; 10 Academic Unit of General Surgery, Department of Biomedical Sciences and Human Oncology, University of Bari “Aldo Moro” Medical School, Bari, Italy; 11 Unit of HPB and Emergency Surgery, General Regional Hospital F. Miulli, Acquaviva delle Fonti, Bari, Italy; 12 Department of Digestive Oncologic and Metabolic Surgery, Institut Mutualiste Montsouris, Paris Descartes University, Paris, France; 13 Cambridge University Hospitals NHS Foundation Trust, Addenbrooke’s Hospital, Cambridge Biomedical Campus, Cambridge, UK; 14 Gastroenterology and Endoscopy Unit, University Hospital of Parma, University of Parma, Parma, Italy; 15 Department of General and Digestive Surgery, Hospital Universitario Doctor Peset, Valencia, Spain; 16 Department of Surgery, St. Vincent’s University Hospital, Elm Park, Dublin 4, Ireland; 17 Department of Emergency and Trauma Surgery, Parma University Hospital, Parma, Italy

**Keywords:** Splenic flexure carcinoma, Emergency surgery, Mortality, Survival, Colectomy, Cancer

## Abstract

**Background:**

The effectiveness of surgical treatment for splenic flexure carcinomas (SFCs) in emergency settings remains unexplored. This study aims to compare the perioperative and long-term outcomes of different alternatives for emergency SFC resection.

**Method:**

This multicenter retrospective study was based on the *SFC Study Group* database. For the present analysis, SFC patients were selected if they had received emergency surgical resection with curative intent between 2000 and 2018. Extended right colectomy (ERC), left colectomy (LC), and segmental left colectomy (SLC) were evaluated and compared.

**Results:**

The study sample was composed of 90 SFC patients who underwent emergency ERC (*n* = 55, 61.1%), LC (*n* = 18, 20%), or SLC (*n* = 17, 18.9%). Bowel obstruction was the most frequent indication for surgery (*n* = 75, 83.3%), and an open approach was chosen in 81.1% of the patients. A higher incidence of postoperative complications was observed in the ERC group (70.9%) than in the LC (44.4%) and SLC groups (47.1%), with a significant procedure-related difference for severe postoperative complications (Dindo-Clavien ≥ III; adjusted odds ratio for ERC vs. LC:7.23; 95% CI 1.51-34.66; *p* = 0.013). Anastomotic leakage occurred in 8 (11.2%) patients, with no differences between the groups (*p* = 0.902). R0 resection was achieved in 98.9% of the procedures, and ≥ 12 lymph nodes were retrieved in 92.2% of patients. Overall and disease-free survival rates at 5 years were similar between the groups and were significantly associated with stage pT4 and the presence of synchronous metastases.

**Conclusion:**

In the emergency setting, ERC and open surgery are the most frequently performed procedures. ERC is associated with increased odds of severe postoperative complications when compared to more conservative SFC resections. Nonetheless, all the alternatives seem to provide similar pathologic and long-term outcomes, supporting the oncological safety of more conservative resections for emergency SFCs.

## Introduction

The surgical treatment of splenic flexure carcinoma (SFC) has been traditionally neglected in the literature, mainly because of its relatively low incidence, as it represents only 3 to 5% of all colonic cancers [1–4]. In recent years, however, several studies have questioned which type of resection would provide the best surgical and oncological outcomes in patients with SFCs [1, 5–13]. Despite a substantial lack of standardization regarding the nomenclature of the different surgical alternatives to resect SFCs, three main surgical procedures are performed, namely, extended right colectomy (ERC), left colectomy (LC), and segmental left colectomy (SLC) [5, 14, 15]. All these procedures are considered alternatives for curative resections of these tumors, which are located on the border between the right and left colon and have dual lymphatic drainage toward the superior and inferior mesenteric vessels [16]. Indeed, two recent meta-analyses concluded that no procedure-related difference exists in terms of postoperative morbidity, mortality, lymph node yield, and patient survival [14, 16]. Consequently, a more conservative SLC, for a long time considered oncologically inadequate [17], is considered a safe and effective option for the treatment of SFCs [5, 18].

In contrast, the outcomes of these different resections when performed in acute clinical situations remain substantially unexplored. Although SFCs have been associated with a poorer prognosis than cancers from other colonic subsites due to the high risk of bowel obstruction [3, 19, 20], the number of emergency cases reported in the literature is limited. Indeed, no prior study has considered emergency SFC resections exclusively. Previous works investigated either samples of elective SFC cases only [5, 6, 9, 11] or mixed populations of patients, including both elective and emergency operations, none of which provided subgroup analyses on emergency cases [7, 8, 10, 18, 21]. In these studies, emergency patients represented 5.7 to 50% of the samples, with an absolute number of cases ranging from 34 to 75 patients [2, 7, 8, 10, 18, 21]. The authors, however, consistently reported that ERC was the preferred approach to treat SFCs presenting as intestinal obstruction [14].

The present study aims to compare the perioperative and long-term outcomes of the different surgical alternatives for SFC resection in emergency settings using the *SFC Study Group* database [5].

## Methods

### Study design

The present study was designed as an ancillary analysis by the *SFC Study Group.* As previously described [5], the *SFC Study Group* was established in March 2017 and involved 11 European surgical units from tertiary care centers to compose a multicenter database on SFC patients undergoing surgery in both elective and emergency settings between January 2000 and January 2018. The participating centers were University Hospital Henri Mondor of Creteil, France; Institute Mutualiste Montsouris of Paris, France; University Hospital of Clermont-Ferrand, France; University Hospital of Geneva, Switzerland; Vall d’Hebron University Hospital of Barcelona, Spain; Viladecans Hospital of Barcelona, Spain; University Hospital Dr. Peset of Valencia, Spain; Portsmouth Hospitals NHS Trust, UK; Addenbrooke’s Hospital of Cambridge, UK; St Vincent’s University Hospital of Dublin, Ireland; University Medical School “Aldo Moro” of Bari, Italy; and Bufalini Hospital of Cesena, Italy.

Anonymous patient data were retrieved from each local database and merged into a common database that was centralized by the leading center and further set for the present statistical analyses. Due to the retrospective design of the study, which was conducted exclusively using patient records, no institutional review board approval was required. All personal data were managed in conformity to the principles declared to the National Commission for Data Protection and Liberties. The study was reported following the recommendations listed in the STROBE checklist for cohort and case–control studies [22].

### Study population

For the present analyses, SFC patients were selected if they met the following criteria: (1) age > 18 years; (2) colon cancer located at the splenic flexure (i.e., 10 cm proximal toward the transverse colon or 10 cm distal to the descending colon [2, 5, 17]) as assessed by preoperative computed tomography (CT) and confirmed during surgery and at the pathologic report [2, 6, 17]; (3) any AJCC TNM stage [23]; (4) obstructive or perforated neoplasm requiring emergency surgery; (5) curative-intent surgical resection; and (6) one- or two-stage surgery (via temporary stoma). Patients with synchronous colonic carcinomas, untreatable metastatic disease, and polyposis coli were excluded. Emergency surgery was defined as an unplanned procedure performed within 48 h of hospital admission [10, 18]. As previously described [5], three types of surgical procedures were performed for SFC resection: ERC, LC, and SLC [2, 5, 14, 15]. Both laparoscopic and open procedures were considered. Interventions were carried out by general and colorectal surgeons. Patients were treated and followed-up according to national protocols [5]. Adjuvant chemotherapy was generally indicated in patients with TNM III/IV tumors or in those presenting cancers with unfavorable histopathological features.

### Study outcomes

The study outcomes were the same as those reported previously on the elective management of SFCs [5] and included intraoperative variables (e.g., operative time, blood loss), postoperative variables (e.g., postoperative morbidity and mortality, length of hospital stay), quality of the surgical resection (e.g., resection margin status, number of retrieved lymph nodes), and overall survival (OS) and disease-free survival (DFS) up to 5 years. Conversion was defined as a premature interruption of the laparoscopic approach before the resection phase was concluded [24, 25]. Postoperative morbidity and mortality were defined as events that occurred during the hospital stay or within 90 days after surgery. Postoperative complications were graded according to the Dindo-Clavien classification [26], with grades of III or more identifying severe complications. Postoperative ileus was defined as the absence of bowel movements or flatus associated with intolerance of oral intake lasting more than 3 days postoperatively [27, 28]. Resections were classified as R0 when a complete removal of the tumor with free resection margins and no peritoneal spread were objectivized macro- and microscopically.

### Statistical analysis

Demographics, clinical characteristics, and study outcomes were compared between the 3 procedures applied to resect SFCs using the chi-squared test for categorical variables and Kruskal-Wallis tests for continuous variables. A multivariate analysis was performed by including demographic, preoperative, and oncological variables that reached a *p* value < 0.1 in the univariate analysis. Adjusted *p* values were reported for the overall comparisons, and whenever significant, two-by-two group comparisons were also explored. Bonferroni correction was applied. Adjusted odds ratios (AORs) were calculated and presented with the 95% confidence interval (CI).

The Kaplan-Meier method was used for the survival analyses, and the log-rank (Mantel-Cox) test was applied for group comparisons. OS was defined as the time from surgery to disease-related death and was censored at the last follow-up date if no events occurred. DFS was defined as the time from surgery to disease recurrence and was censored at the last follow-up date if no events occurred. Univariate and multivariate Cox proportional hazards regression models were used to identify independent predictors of survival.

Statistics were carried out with SPSS (Statistical Package for the Social Sciences, IBM SPSS Statistics, Version 23 for Macintosh; IBM Corp., Armonk, NY, USA). A *p* value < 0.05 was considered statistically significant.

## Results

**Fig. 1:**
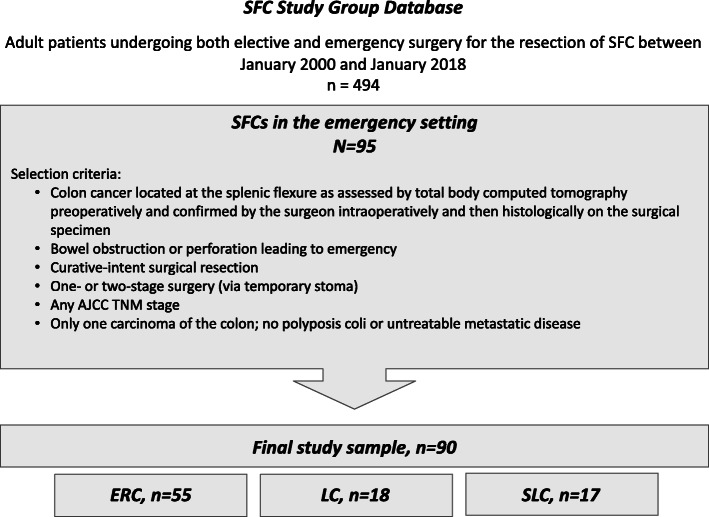
Study sample selection flowchart from the *SFC Study Group* database. ERC, extended right colectomy; LC, left colectomy; SLC, segmental left colectomy for splenic flexure carcinomas (SFCs)

The original *SFC Study Group* database included 494 SFC patients. Of these, 95 patients underwent emergency surgery. Five patients were excluded because they did not meet all the inclusion criteria or due to missing data. Finally, the present study sample was composed of 90 patients who underwent emergency ERC (*n* = 55, 61.1%), LC (*n* = 18, 20%), or SLC (*n* = 17, 18.9%) (Fig. 1). SFCs were located at the splenic flexure (*n* = 56, 62.2%), up to 10 cm proximal toward the transverse colon (*n* = 17, 18.9%), or up to 10 cm distal toward the descending colon (*n* = 17, 18.9%), without differences between the 3 surgical procedure groups (*p* = 0.389). For the majority of patients, surgery was indicated due to bowel obstruction (*n* = 75, 83.3%), whereas the remaining presented with a perforation (*n* = 15, 16.7%). No procedure-related differences were observed (*p* = 0.451). No endoscopic colonic stent was used as a bridge to surgery. Overall, 73 patients (81.1%) underwent open surgery; a higher frequency of laparoscopy was observed for LC and SLC procedures than ERC, but the difference was not statistically significant (*p* = 0.051). The majority of interventions (57.8%) were performed by colorectal surgeons without procedure-related differences (*p* = 0.379). Demographic, clinical, preoperative, and histopathological characteristics for the total study sample and by surgical procedure are displayed in Table 1.

**Table 1:** Demographic, clinical, imaging, and histological/oncological characteristics of SFC patients operated on by ERC, LC, and SLC

	Total sample (*n* = 90)	ERC (*n* = 55)	LC (*n* = 18)	SLC (*n* = 17)	*P* value
*Demographic and clinical variables*					
Gender (M/F) [*n*]	53/37	33/22	12/6	8/9	0.482
Age (year) [median (range)]	71 (33-94)	73 (38-94)	61.5 (33-90.7)	74.7 (43-84.3)	**0.044**
Age > 75 (year) [*n* (%)]	34 (37.8)	22 (40)	4 (22.2)	8 (47.1)	0.274
BMI (kg/m^2^) [median (range)]	25.8 (14-34)	25.3 (14-34)	22 (17-32.3)	26.1 (21-34)	0.082
Obesity (BMI ≥ 30 kg/m^2^) [*n* (%)]	16 (17.8)	10 (18.2)	2 (11.1)	4 (23.5)	0.626
ASA score [*n*%]					**0.043**
• I-II	42 (46.7)	21 (38.2)	13 (72.2)	8 (47.1)	
• III-IV	48 (53.3)	34 (61.8)	5 (27.8)	9 (52.9)	
Pre-operative leukocytes (10^9^/L) [mean (SD)]	10.96 (4.73)	11.5 (5.21)	10.99 (3.60)	8.95 (3.48)	0.198
Weight loss > 10% [*n* (%)]	25 (27.8)	16 (29.1)	6 (33.3)	3 (17.6)	0.550
Pre-operative serum CEA (U/mL) [median (range)]	33.65 (0.4-275.5)	33.65 (1.10-244.7)	54.7 (0.4-275.5)	23 (1.4-92.9)	0.241
Comorbidity (> 1) [*n* (%)]	38 (42.2)	25 (45.5)	6 (33.3)	7 (41.2)	0.662
Diabetes [*n* (%)]	14 (15.6)	10 (18.2)	2 (11.1)	2 (11.8)	0.689
Cardiopulmonary diseases [*n* (%)]	57 (63.3)	32 (58.2)	12 (66.7)	13 (76.5)	0.372
Kidney failure [*n* (%)]	6 (6.7)	4 (7.3)	1 (5.6)	1 (5.9)	0.958
Neurocognitive disorders [*n* (%)]	14 (15.6)	7 (12.7)	2 (11.1)	5 (29.4)	0.213
Smoking [*n* (%)]	30 (33.3)	20 (36.4)	6 (33.3)	4 (23.5)	0.618
Indication for surgery [*n* (%)]					0.451
• Colonic obstruction	75 (83.3)	48 (87.3)	14 (77.8)	13 (76.5)	
• Colonic perforation	15 (16.7)	7 (12.7)	4 (22.2)	4 (23.5)	
Surgical approach [*n* (%)]					
• Laparoscopy	17 (18.9)	6 (10.9)	6 (33.3)	5 (29.4)	0.051
• Open surgery	73 (81.1)	49 (89.1)	12 (66.7)	12 (70.6)	
Previous abdominal surgery [*n* (%)]					
• Laparoscopy	6 (6.7)	4 (7.3)	0	2 (11.8)	0.363
• Open surgery	20 (22.2)	11 (20)	3 (16.7)	6 (35.3)	0.340
One- or two-stage surgery [*n* (%)]					0.236
• One-stage surgery with primary anastomosis	70 (77.8)	46 (83.6)	12 (66.7)	12 (70.6)	
• Two-step procedure by temporary ostomy	20 (22.2)	9 (16.4)	6 (33.3)	5 (29.4)	
Simultaneous splenectomy [*n* (%)]	8 (8.9)	5 (9.1)	3 (16.7)	0	0.222
*Preoperative imaging assessment on CT-scan*					
Tumor size (largest dimension, cm) [mean (SD)]	4.44 (2.01)	4.24 (1.67)	5.55 (2.92)	3.93 (1.46)	0.070
Peri-colic nodal involvement [*n* (%)]	40 (44.4)	21 (38.2)	9 (50)	10 (58.8)	0.283
Patients with suspected extra-colic organs involved [*n* (%)]	5 (5.6)	0	5 (27.8)	0	**< 0.0001**
Suspected synchronous metastasis [*n* (%)]	16 (17.8)	11 (20)	2 (11.1)	3 (17.6)	0.693
*Histological/oncological variables*					
Stage of disease AJCC [*n* (%)]					0.490
• I	5 (5.6)	5 (9.1)	0	0	
• II	34 (37.8)	21 (38.2)	8 (44.4)	5 (29.4)	
• III	40 (44.4)	22 (40)	9 (50)	9 (52.9)	
• IVa	11 (12.2)	7 (12.7)	1 (5.6)	3 (17.6)	
Vascular invasion [*n* (%)]	27 (30)	15 (27.3)	6 (33.3)	6 (35.3)	0.772
Lymphatic invasion [*n* (%)]	33 (36.7)	17 (30.9)	8 (44.4)	8 (47.1)	0.360
Perineural invasion [*n* (%)]	24 (26.7)	11 (20)	9 (50)	4 (23.5)	**0.042**
Tumor size-largest dimension (cm) [mean (SD)]	4.6 (1.73)	4.56 (1.94)	5.19 (1.35)	4.07 (1.19)	**0.039**
Tumor grade [*n* (%)]					**0.024**
• Well differentiated	26 (28.9)	21 (38.2)	3 (16.7)	2 (11.8)	
• Moderately differentiated	48 (53.3)	22 (40)	12 (66.7)	14 (82.4)	
• Poorly differentiated	16 (17.8)	12 (21.8)	3 (16.7)	1 (5.9)	
Adjuvant treatment [*n* (%)]	44 (48.9)	24 (43.6)	11 (61.1)	9 (52.9)	0.408

Significant *p* values are indicated in bold

*AJCC* American Joint Committee on Cancer, *ASA* American Society of Anesthesiology, *BMI* body mass index, *CEA* carcinoembryonic antigen, *CT* computed tomography, *ERC* extended right colectomy, *LC* left colectomy, *SLC* segmental left colectomy, *SFC* splenic flexure cancer

### Comparisons between the three surgical procedures for SFCs

No procedure-related differences were observed except for age, ASA score, and suspected extracolic organ involvement upon CT scan (Table 1). In the histopathological analysis, patients in the LC group had the largest tumors and presented a higher prevalence of perineural invasion and moderately or poorly differentiated cancers. Regarding the intra- and postoperative outcomes, a higher incidence of postoperative complications was observed in the ERC group (70.9%) than in the LC (44.4%) and SLC groups (47.1%); however, a significant procedure-related difference was noted only when considering severe postoperative complications (Dindo-Clavien ≥ III) (*p* = 0.011). A similar trend was also observed for the time to return to regular diet, which was longer for patients operated on by ERC (*p* = 0.054) (Table 2). In the adjusted model, the only significant between-group difference was observed for the rate of severe postoperative complications; ERC was associated with an increased risk of Dindo-Clavien ≥ III, particularly when compared to LC (AOR 7.23; 95% CI 1.51-34.66; *p* = 0.013).

**Table 2:** Operative and postoperative outcomes in SFC patients (*n* = 90) operated on by ERC, LC, and SLC

Variables	ERC (*n* = 55)	LC (*n* = 18)	SLC (*n* = 17)	*p* value	Adjusted *p* value
Operative time (min) [mean (SD)]	212.71 (70.91)	206.11 (47.67)	215.06 (67.37)	0.986	0.415
Conversion to laparotomy^b^ [*n* (%)]	0	1 (16.7)	1 (20)	0.531	0.102
Operative blood loss (mL) [mean (SD)]	202.18 (153.43)	213.89 (193.57)	194.12 (151.70)	0.951	0.994
Number of intraoperative transfused patients [*n* (%)]	4 (7.3)	4 (22.2)	3 (17.6)	0.183	0.077
Intraoperative complication [*n* (%)]	10 (18.2)	0	2 (11.8)	0.141	0.997
Patients with post-operative complication [*n* (%)]	39 (70.9)	8 (44.4)	8 (47.1)	0.057	0.064
Anastomotic leakage^a^ [*n* (%)]	6 (13)	1 (8.3)	1 (8.3)	0.892	0.902
Postoperative ileus [*n* (%)]	9 (16.4)	2 (11.1)	1 (5.9)	0.514	0.393
Severe postoperative complications (Dindo-Clavien ≥III) [*n* (%)]	27 (49.1)	2 (11.1)	5 (29.4)	**0.011**	0.032*
Time to flatus [mean (SD)]	6.15 (5.08)	4.67 (2.08)	4.75 (3.09)	0.288	0.455
Return to regular diet [mean (SD)]	9.58 (6.24)	6.89 (3.37)	7.5 (6.5)	0.054	0.585
Hospital stay, days [mean (SD)]	17.62 (14.7)	14.40 (2.29)	17.75 (20.57)	0.735	0.465
Mortality at 90 days [*n* (%)]	8 (14.5)	1 (5.6)	1 (5.9)	0.430	0.677
Readmission within 60 days [*n* (%)]	4 (8.5)	1 (5.6)	0	0.477	0.987
Positive resection margin [*n* (%)]	1 (1.8)	0	0	0.725	1
Number of lymph nodes harvested [mean (SD)]	24.8 (12.97)	29.89 (11.53)	21.06 (6.89)	0.064	0.189
• ≥12 lymph nodes [*n* (%)]	49 (89.1)	18 (100)	16 (94.1)	0.308	0.250

Significant *p* values are indicated in bold

*ERC* extended right colectomy, *LC* left colectomy, *SLC* segmental left colectomy, *SFC* splenic flexure cancer

^*^Adjusted odds ratio and 95% confidence interval for pairwise comparisons:

ERC vs. LC = 0.013; OR: 7.23 (95% CI: 1.51-34.66)

ERC vs. SLC = 0.212; OR: 2.12 (95% CI: 0.65-6.91)

LC vs. SLC = 0.334; OR: 0.24 (95% CI: 0.01-4.25)

^a^Calculated for patients with primary anastomosis

^b^Calculated for patients operated on by laparoscopy

**Fig. 2:**
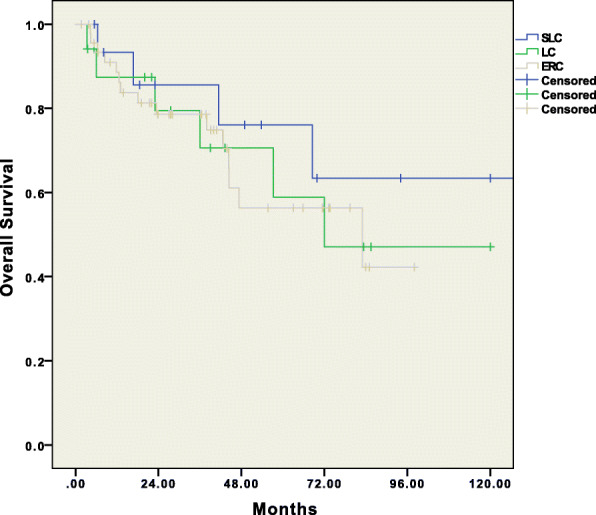
Survival analyses (Kaplan-Meier method) for overall survival for SFC patients operated on by ERC (extended right colectomy), LC (left colectomy), and SLC (segmental left colectomy)

**Fig. 3:**
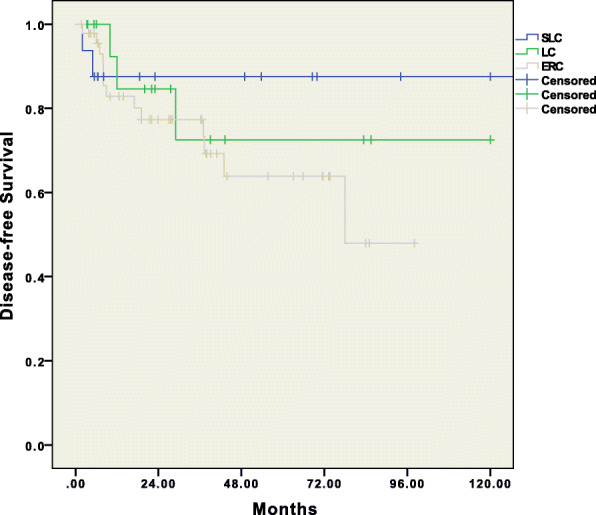
Survival analyses (Kaplan-Meier method) for disease-free survival for SFC patients operated on by ERC (extended right colectomy), LC (left colectomy), and SLC (segmental left colectomy)

Overall, 2/17 (11.7%) patients required conversion from laparoscopy to open surgery, one in the LC group and one in the SLC group. Reasons for conversion included technical difficulties due to bulky tumors (1) and difficult exposure and inadequate visualization due to tumor fixation (1). Anastomotic leakage occurred in 8 (11.2%) patients, with no differences between groups (*p* = 0.902). Seventeen patients (18.9%) required reoperation for the following reasons: anastomotic leakage (8), postoperative evisceration (2), hemoperitoneum due to splenic bleeding (3), explorative laparotomy for suspected anastomotic leakage that was not objectivized (2), peritonitis due to bowel perforation (1), and peritoneal lavage and drainage for pancreatic fistula (1). No group difference was observed (*p* = 0.802). Overall mortality within 90 days was 11.1%. R0 resection was achieved in 98.9% of the patients, and at least 12 lymph nodes were retrieved in 92.2% of resections, without significant procedure-related differences (Table 2). All patients who received a temporary stoma underwent stoma closure within 4 months of SFC surgery. The overall mean follow-up time was 41.59 (± 33.48) months, with no group differences (*p* = 0.406). The OS and DFS are shown in Figs. 2 and 3, respectively.

The 1-, 2-, and 5-year OS rates were 86.2%, 78.6%, and 62.6%, respectively, for the ERC group; 87.4%, 79.4%, and 58.9%, respectively, for the LC group; and 93.3%, 77%, and 63.4%, respectively, for the SLC group (*p* = 0.697). The 5-year OS for the entire study sample was 58.4%.

Overall, 18 patients (22.5%) developed cancer recurrence: 13 (27.7%) in the ERC group, 3 (17.6%) in the LC group, and 2 (12.5%) in the SLC group (*p* = 0.394). Of these, 5 patients (6.2%) developed local recurrence, and 15 patients (18.7%) developed distant metastases, such as isolated liver metastasis (7), isolated pulmonary metastasis (3), liver and pulmonary metastasis (1), peritoneal carcinomatosis (2), and systemic metastatic disease (2), with no differences among the groups (*p* = 0.563). The 1-, 2-, and 5-year DFS rates were 80.3%, 74.3%, and 57.5%, respectively, for the ERC group; 84.6%, 75.2%, and 60.2%, respectively, for the LC group; and 78.7%, 69.6%, and 58.2%, respectively, for the SLC group (*p* = 0.402). The 5-year DFS for the entire study sample was 67.3%.

Stage pT4 and the presence of synchronous metastases were found to be independent predictors for OS and DFS (Table 3).

**Table 3:** Univariate and multivariate Cox regression hazard analyses of predictors of overall and disease-free survival

	Whole sample (***n*** = 80*)							
	Overall survival				Disease-free survival			
Variables	Univariate analysis		Multivariate analysis		Univariate analysis		Multivariate analysis	
	***p*** value	HR (95% CI)	***p*** value	Adjusted HR (95% CI)	***p*** value	HR (95% CI)	***p*** value	Adjusted HR (95% CI)
**Male vs. female**	0.666				0.375			
**Age > 75 year**	0.493				0.312			
**Comorbidity > 1**	0.742				0.085			
**Open vs. laparoscopic approach**	0.880				0.966			
**ERC vs. LC + SLC**	0.596				0.200			
**Synchronous metastasis**	**< 0.0001**	**13.37 (5.34-33.45)**	**< 0.0001**	**11.39 (4.46-29.11)**	**< 0.0001**	**23.92 (6.98-81.97)**	**> 0.0001**	**27.72 (7.17-87.15)**
**pT4 vs. pT1-3**	**0.007**	**2.95 (1.33-6.54)**	**0.046**	**2.31 (1.17-5.27)**	**0.024**	**2.92 (1.15-7.44)**	**0.028**	**3.04 (1.12-8.26)**
**pN+ vs. pN0**	0.146				0.059			
**Harvested lymph nodes < 12**	0.814				0.976			
**Adjuvant chemotherapy**	0.690				0.111			
**Dindo-Clavien ≥ III**	0.452				0.411			
**Obstruction vs. perforation**	0.935				0.356			

HR < 1 indicates an improvement in survival (positive prognostic factor); HR > 1 indicates a worse survival (negative prognostic factor)

Significant *p* values are in bold characters

*HR* hazards ratio, *CI* for confident interval, *AJCC* American Joint Committee on Cancer, *SFC* splenic flexure cancer

*After removing patients deceased within 90 days post-surgery (*n* = 10)

## Discussion

The present multicenter retrospective study is the first, to our knowledge, to describe and compare the outcomes of the different surgical resections performed for SFCs presenting as a surgical emergency. Our results confirm that ERC and open surgery are the most frequently performed procedures in this clinical situation. Moreover, ERC appears to be associated with higher rates of severe postoperative complications. Comparable short- and long-term oncologic outcomes are observed between ERC, LC, and SLC, supporting the feasibility and safety of more conservative SFC resections in emergency settings.

The choice of which procedure to perform for cancers located at the splenic flexure remains essentially based on the surgeon’s experience and preference. In the setting of elective surgery, accumulating evidence consistently shows no difference in terms of oncological outcomes and survival between ERC, LC, and SLC [14, 16]; however, some previous comparative studies reported that extensive SFC resections are associated with higher rates of postoperative complications, particularly postoperative ileus [5, 6], and longer recovery times [5], suggesting a short-term benefit in performing more limited SFC resections (LC and SLC) [5, 6, 11, 18].

For emergency surgery, however, a few studies did not provide any subgroup analysis separately assessing the outcomes for the different types of SFC resection performed as emergency and elective surgery. The main reason for this lack of evidence could be related to the very low incidence of emergency SFCs. To date, all the available evidence for SFC resection is supported by retrospective evaluations of single or multicenter studies spanning very long time frames (up to 20 years) to have a relatively large number of cases. The *SFC Study Group* database included 494 SFC patients, of whom 95 (19.2%) were treated emergently. This percentage is higher than those reported in other multicenter studies with large sample sizes. Degiuli et al. [18] analyzed a nationwide population of 1304 SFC patients, only 5.7% of which underwent emergency surgery over the considered period of 10 years. Binda et al. [21] included 16.6% of emergency cases in their multicenter study sample of 324 SFC patients operated on between 2004 and 2015 and found a significantly higher frequency of ERC than LC as emergency procedures (22.8% vs. 10.8%, respectively). Moreover, the authors observed higher mortality, overall morbidity, and surgical site infections for emergency procedures, disregarding the type of resection (i.e., ERC+LC). Importantly, all emergent procedures in the study were performed by open surgery [21]. Martin Arévalo et al. [8] performed a propensity score matching study including 52 (30.5%) emergency surgeries (26 ERC, 17 LC, and 9 SLC), which were analyzed together with elective procedures. Therefore, the present study describes the largest sample of emergency SFC resections published so far.

Consistent with the previous literature, ERC was the most frequently performed procedure, representing 61% of all emergency resections. Similarly, open surgery was chosen in more than 80% of cases, which is the main difference compared to the elective SFC resections of the *SFC Study Group* database published previously by de’Angelis et al. [5] in which 74.4% were laparoscopic procedures. This difference can be easily linked to the clinical scenario in which the surgeon has to choose the surgical approach facing an SFC presenting as bowel obstruction (83% of cases) or perforation. ERC and open surgery are usually preferred because they may be technically easier in the presence of proximal colonic distension or may be mandatory due to concerns about the viability of the cecum [8, 14]. Indeed, the experience of the surgeon on call, who may not always be a specialized colorectal and/or laparoscopic surgeon, could have an impact on this choice [10, 21]. Binda et al. [21] observed some differences in terms of the choice of the surgical procedure and approach when comparing general surgeons vs. colorectal surgeons, but only for elective surgery. In the present study, almost 60% of the procedures were performed by specialized colorectal surgeons, and no difference was observed between the ERC, LC, and SLC groups. The type of resection and the surgeon’s experience have also been related to some surgical outcomes, such as postoperative complications, R0 resection, and lymph node yield. The present findings suggest that ERC may be associated with increased odds of developing severe postoperative complications when compared with LC and SLC; however, similar and favorable pathologic outcomes were observed for all SFC resection alternatives, with an R0 obtained in almost 99% of cases and more than 12 lymph nodes harvested in 92% of patients. Importantly, these data confirm the findings already reported for elective ERC, LC, and SLC [5, 10, 14, 16], supporting the oncological safety of more conservative SFC resections in emergency settings. Patient survival rates were comparable among the ERC, LC, and SLC groups, with a global sample 5-year OS of 58.4%. These rates are inferior to those reported in studies concerning elective SFC resections or mixed populations [14, 16], but they are comparable to the study by Odermatt et al. [10] that considered the highest percentage of emergency surgery in their sample (50% of patients). Poorer OS and DFS may be expected in emergency patients who present with significant comorbidities, unstable conditions, or advanced tumor stage at diagnosis [10]. The present results, taking into account potential confounders, showed that for patients operated on in emergency settings, predictors for OS and DFS are essentially associated with tumor characteristics, particularly stage pT4 and the presence of synchronous metastasis.

The present retrospective study has some limitations, and these findings should be interpreted and generalized with caution because they are based on a sample of patients operated on in several European referral centers over a long period of time. The potential impact of selection and reporting bias cannot be excluded.

## Conclusion

The present study, based on a relatively large sample of SFC patients undergoing emergency surgery, confirms that ERC and open surgery are the most frequently performed procedures. ERC appears to be associated with a higher incidence of severe postoperative complications, whereas R0 resection, lymph node yield, tumor recurrence, and survival rates are comparable between ERC, LC, and SLC. These findings also support the feasibility and safety of more conservative SFC resections in emergency settings.

## Data Availability

There are no data from individual authors that reach the criteria for availability.

## Abbreviations

ASA: American Society of Anesthesiologists
BMI: Body mass index
CT: Computed tomography
DFS: Disease-free survival
ERC: Extended right colectomy
LC: Left colectomy
OS: Overall survival
SFC: Splenic flexure carcinoma
SLC: Segmental left colectomy
